# Architectural RNA in chromatin organization

**DOI:** 10.1042/BST20191226

**Published:** 2020-09-08

**Authors:** Jitendra Thakur, Steven Henikoff

**Affiliations:** 1 Basic Sciences Division, Fred Hutchinson Cancer Research Center, Seattle, WA 98109, U.S.A.; 2 Howard Hughes Medical Institute, Basic Sciences Division, Fred Hutchinson Cancer Research Center, Seattle, WA 98109, U.S.A.

**Keywords:** architectural RNA, chromatin, compaction, heterochromatin, nuclear bodies, phase separation

## Abstract

RNA plays a well-established architectural role in the formation of membraneless interchromatin nuclear bodies. However, a less well-known role of RNA is in organizing chromatin, whereby specific RNAs have been found to recruit chromatin modifier proteins. Whether or not RNA can act as an architectural molecule for chromatin remains unclear, partly because dissecting the architectural role of RNA from its regulatory role remains challenging. Studies that have addressed RNA's architectural role in chromatin organization rely on *in situ* RNA depletion using Ribonuclease A (RNase A) and suggest that RNA plays a major direct architectural role in chromatin organization. In this review, we will discuss these findings, candidate chromatin architectural long non-coding RNAs and possible mechanisms by which RNA, along with RNA binding proteins might be mediating chromatin organization.

## Introduction

Of the three components of the central dogma (DNA, RNA and Protein), RNA is the most versatile molecule. RNA functions universally as the genetic messenger and as transfer RNAs that deliver amino acids to the site of protein synthesis on the RNA-scaffolded ribosome. In addition, ribozymes catalyze certain specific biochemical reactions, small RNAs (∼20–30 nucleotides) silence gene expression via transcriptional, post-transcriptional as well as chromatin-dependent gene silencing pathways and a functionally diverse class of >200 nucleotides long non-coding RNAs (lncRNAs) play roles in gene expression, genomic imprinting and genome organization [1–6]. Another distinct class of small RNAs (small nuclear RNAs and small nucleolar RNAs) of ∼ 150 nucleotides length, plays important roles in splicing of introns from pre-messenger RNA in the nucleus [7,8]. RNA also acts as the regulator of nuclear and chromatin organization [9]. Furthermore, RNA is the genetic material of retroviruses and other diverse viral clades [10,11].

## RNA as an architectural molecule

RNA is an excellent architectural molecule due to its ability to form extensive secondary structures which provide surfaces for various RNA binding proteins (RBPs) thereby forming RNA–RBP scaffolds. These RNA–RBP architectural scaffolds are well known to form nuclear bodies, such as nucleoli, nuclear speckles and nuclear stress bodies, in the interchromatin space where RNA is highly abundant. Removal of RNA leads to collapse of these nuclear bodies providing clear evidence for the architectural role of RNA in formation of these structures [12–14]. A significant fraction of RNA has also been found to directly interact with chromatin and is proposed to have a structural role in chromatin organization [15–18]. However, unlike the well-established architectural role of RNA for nuclear bodies, its equivalent role in chromatin organization remains unclear. Direct chromatin structural modifiers identified to date are only proteins. Histone proteins package genomic DNA into nucleosomal arrays, which are further organized into more condensed heterochromatin and more open euchromatin by chromatin modifier proteins [19–24]. Heterochromatin protein 1 (HP1) and Polycomb Repressive Complex 1 (PRC1) condense nucleosomal arrays into compact heterochromatin, which limits access to DNA metabolism machineries. In contrast, euchromatic proteins maintain a more accessible chromatin environment that facilitates active transcription of underlying loci.

Here, we will discuss the potential role of RNA in maintaining the native structure of chromatin. RNA was shown to be a structural component of the nuclear matrix, a seemingly filamentous structure that spans the interchromatin space in electron microscopy images. However, the existence of the nuclear matrix as a discrete structure is now considered to have been a fixation artifact, as such structures were not visualized in living cells or using high-resolution cryo-electron tomography [25,26]. In contrast, membraneless nuclear bodies formed by architectural RNA are well characterized and can be cytologically visualized in the interchromatin space [12]. Most of these nuclear bodies are reservoirs of either small nucleolar RNA (snoRNA), transcribed mRNA and/or lncRNA, which maintain the integrity of these bodies by forming multivalent weak electrostatic or hydrophobic interactions with RBPs [13]. For example, associations between lncRNA NEAT1 and RNA-binding motifs containing proteins PSF/SFPQ, P54NRB/NONO, and PSPC1 create dynamic structural scaffolds that form paraspeckles [14,27]. The inducible transcription of NEAT1 followed by the direct visualization of the recruitment of paraspeckle proteins by live cell imaging has revealed that the act of NEAT1 transcription, and not lncRNAs alone, regulates paraspeckle maintenance [28]. Paraspeckles disappear after incubation with RNase A suggesting that the presence of RNA in paraspeckles is essential to maintain paraspeckle structure (DNase I does not affect their structural integrity) [27]. Paraspeckles also disassemble in the absence of active RNA Polymerase II transcription and subsequently reassemble on its restoration, suggesting that their integrity is not only dependent on the presence of RNA but also on RNA production [27]. Similarly, the nucleolus, which is formed by ribosomal RNA (rRNA) and RBPs (e.g. nucleophosmin, fibrillarin etc.), collapses into an irregular structure upon inhibition of transcription or by depletion of RNA [29]. Moreover, tethering RNA found in these nuclear bodies is sufficient to nucleate many of these bodies [12]. Given the structural role of RNA in the formation of nuclear bodies, the term architectural RNA has been attributed to RNAs associated with nuclear bodies [30,31]. Here, we will expand the use of the term architectural RNA to describe all RNA species that act as structural scaffolds for nuclear structures including chromatin.

**Figure 1.**
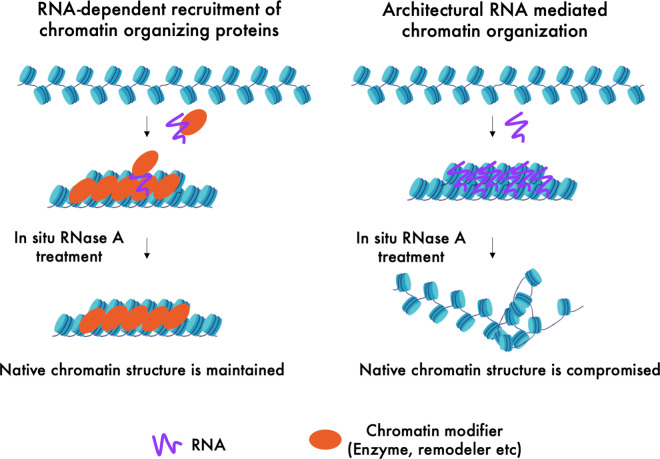
Schematic showing indirect regulatory and direct architectural role of RNA in chromatin organization. RNA is known to recruit chromatin modifiers such as histone modifying enzymes, chromatin remodelers and chromatin compacting proteins (Left). RNA can also act as a tether to fold or compact chromatin by direct interactions (Right). *In situ* RNA depletion by RNase A treatment leads to loss of total nuclear RNA, while keeping the RNA-independent nuclear structures intact. The regions organized into specific chromatin configurations by architectural RNA will lose their structure upon RNA depletion while the regions maintained by chromatin modifiers proteins with not.

A significant fraction of RNA, mostly non-coding, has also been found to associate with chromatin [15–18,32,33]. RNA is the product of transcription, a process that in itself participates in chromatin organization [34]. Moreover, many non-coding RNAs contribute to chromatin organization by recruiting histone methyltransferases and other RBPs that are chromatin remodelers [35] (Figure 1). Dissecting these indirect regulatory roles from the direct architectural role remains a challenge, and as a result, architectural roles of RNA in chromatin organization are often unclear. Furthermore, unlike nuclear bodies, which are mostly filled with RNAs along with their protein binding partners, chromatin is associated with a low amount of RNA per given unit of chromatin (2%–5% of total nucleic acids in chromatin is RNA) [18]. Therefore, probing structural roles of RNA in chromatin organization requires more sensitive assays that can detect subtle architectural features of RNA in chromatin organization with high resolution in intact cells.

## Evidence for structural roles of RNA in chromatin organization

**Figure 2.**
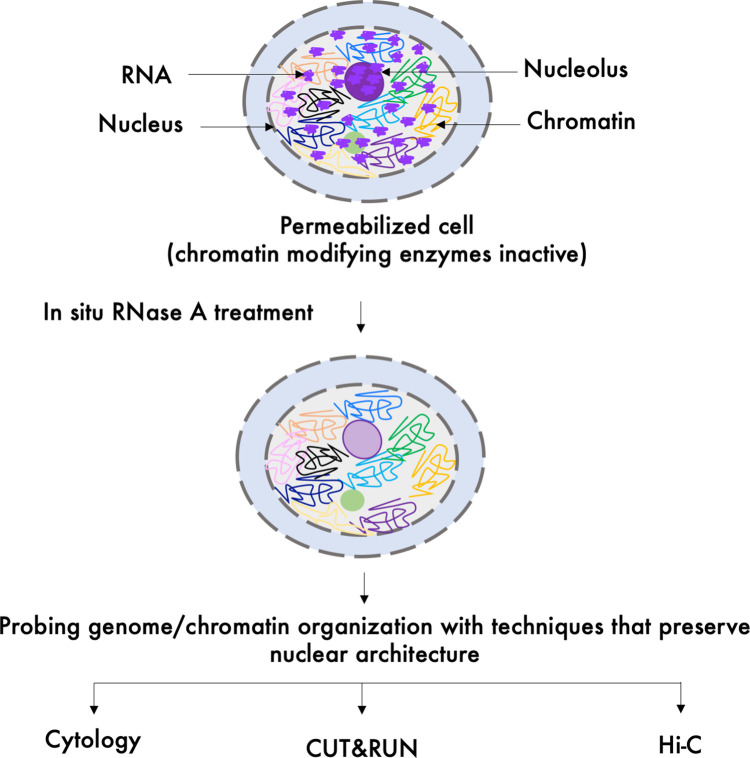
*In situ* RNase A digestion approach to probe RNA's architectural role in chromatin/genome organization. Cell permeabilization with detergents renders chromatin modifying enzymes inactivate. Upon *in situ* RNA depletion by RNase A treatment, histones remain stably bound to DNA in the nucleosomes suggesting that the primary chromatin structure remains unaffected by RNase A treatment [39]. However, the higher order chromatin structure may be affected by the loss of RNA. Whether or not architectural RNA maintains the higher order organization of nuclear structures including chromatin, can be investigated by *in situ* techniques such as cytological visualization, Cleavage Under Targets and Release Using Nuclease (CUT&RUN) [40] and Hi-C [41], in the control and RNase A treated cells.

Each of the studies that have addressed the architectural contribution of RNA in chromatin integrity by either cytological visualization or other *in situ* assays rely on *in situ* RNA depletion using RNase A (Figure 2) [36–38]. RNase A digestion depletes total RNA, which limits further investigation of the role of specific RNAs in chromatin organization. However, *in situ* RNase A digestion provides a powerful system to identify an architectural RNA component in chromatin structure as it rules out indirect contributions of RNA by recruiting chromatin modifying proteins (e.g. histone methyltransferases, chromatin remodelers etc.), which become inactive after cells have been permeabilized with a detergent. In addition, both cytological visualization and *in situ* conformation capture assays (discussed below) preserve overall nuclear structures and can be analyzed in presence or absence of RNA.

One of the earliest reports of an architectural role of RNA came from observing RNase A treated Dinoflagellate cells/nuclei [40]. Dinoflagellate chromosomes are seen as condensed helical structures [40]. Cytological visualization of RNase A treated cells revealed decondensation, stretching and unwinding leading to irregular structures at all levels of chromosome organization [40]. Subsequently, visualization of electron micrographs of RNase A digested HeLa nuclei revealed that the global chromatin, seen as uniformly distributed granules in control cells, collapses into large clumps that fall either onto the nuclear lamina or the nucleolus [36]. This study led to the proposal that RNA is a structural component of the nuclear matrix and may contribute to higher order chromatin structure [36]. The first evidence for a structural role of RNA in maintaining a specific chromatin domain came from visualization of pericentric H3K9me-marked heterochromatin in RNase A treated mouse cells. Mouse pericentric heterochromatin domains are organized as large clusters called chromocenters around the nucleolus [42]. Cytological visualization of mouse nuclei stained using antibodies raised against a branched lysine-9 dimethylated H3 amino terminus peptide revealed a loss of the signals for branched chain epitopes upon *in situ* RNase A treatment as compared with the control untreated cells [37]. The four H3K9 dimethylated peptide ‘fingers’ mimicked the 3-D interaction points within a H3K9me2 heterochromatin domain. Addition of purified cellular RNA to RNase A treated cells restored H3K9me2 clusters, suggesting that the mode of action of architectural RNA is simple and direct [37]. Binding of H3K9me3 histone methyltransferase, SUV39H1, to its target site was lost upon RNase A treatment as well as in nucleic acid binding mutants of SUV39H1 [43]. SUV39H1 binds to nucleic acids with a higher binding affinity for RNA than DNA independent of its binding to H3K9me3 [44]. These findings suggest that direct binding to architectural RNA may help retain histone modifying enzymes onto the chromatin template.

Possible architectural roles of specific transcripts have also been investigated for the binding of chromatin components at centromeres, DNA loci that mediate chromosome segregation by assembling a multproteinaceous structure called kinetochore, which then binds to spindles [45,46]. Centromeric chromatin comprises nucleosomes containing the centromere-specific histone variant CENP-A, which are tightly associated with other key centromeric DNA binding proteins CENP-B, CENP-C and CENP-T [47,48]. Centromeric DNA consists of megabase long arrays of tandem repeats called α-satellites, which transcribe into non-coding mature RNA that are physically associated with centromeric chromatin [49,50]. Targeted deletion of α-satellite transcripts results in a loss of CENP-A and CENP-C in cis [49]. Interestingly, *in situ* depletion of RNA by RNase A treatment reduces centromere binding of CENP-C significantly while leaving CENP-A levels unaffected [51]. These results suggest the presence of an architectural RNA component of centromeric chromatin. In addition, the finding that *in vivo* and not *in situ* RNA depletion alters CENP-A binding suggests that α-satellite transcripts might play both architectural and regulatory roles.

Recently, the Hi-C *in situ* chromosome conformation capture technique has been applied to test for a chromatin architectural role for RNA. Hi-C captures 3-D interactions by ligating proximal genomic regions and can be combined with *in situ* RNase digestion to probe the effect of total RNA depletion on genome organization [52]. Hi-C of control cells detected genomic compartments (compartment A corresponding to euchromatin and compartment B corresponding to heterochromatin) which are further subdivided into distinct long-range topologically associated domains (TADs) that define the set of interactions within a given region [41,53]. Hi-C analysis revealed that while TAD signals remained unaffected, compartment B signals were reduced upon RNA depletion [38]. These results suggest that 3-D genomic interactions between heterochromatic regions are maintained by architectural RNA.

## Candidate architectural lncRNAs involved in chromatin organization

Although a small subset of total lncRNAs are known to play essential roles, many of them are involved in chromatin regulation by recruiting chromatin modifying proteins [54,55] (Table 1). LncRNAs exhibit properties that make them potential candidates for acting as architectural elements for chromatin organization [6]. RNA forms secondary structures that provide unique domains for interaction with specific proteins and other RNA molecules. A single lncRNA can act as an RNA scaffold either by interacting with multiple copies of the same protein or several different proteins at once [56–58].

**Table 1:** Long non-coding RNAs involved in chromatin organization

LncRNAs	Role in chromatin structure/function	References
XIST	- Localizes to the entire inactive X chromosomes and recruits machineries for imparting repressive chromatin marks.	[79]
KCNQ1OT1	- Involved in bidirectional silencing of genes in the Kcnq1 domain.	[80]
	- Interacts with the H3K9- and H3K27-specific histone methyltransferases G9a and the PRC2 complex, respectively in a lineage-specific manner in the placenta.
AIR	- Controls the Igf2r imprinting cluster in mouse by recruiting the G9a histone methyltransferase to silence target genes.	[81]
HOTAIR	- Transcribes from the HOXC locus on human Chr12 and represses transcription in trans across 40 kilobases of the HOXD locus on Chr2.	[66,82]
	- Binds to the histone methyltransferase PRC2 complex and the histone demethylase LSD1/CoREST/REST complex at its 5′ domain 3′ ends, respectively.
MALAT1	- Localizes to nuclear speckles and interacts with pre-mRNA splicing factors and active chromatin	[69]
NEAT1	- Forms paraspeckles by interacting with RNA binding proteins.	[14,69]
SAT III	- Upon stress transcribes from pericentric repeats and forms nuclear stress bodies.	[83]
	- Recruits RNA processing factors to nuclear stress bodies.
COLDAIR	- Represses a floral repressor FLOWERING LOCUS C (FLC) by interacting with PRC2 during vernalization in *Arabidopsis thaliana*.	[84]
roX1 and roX2	- Recruit the dosage-compensation complex on the male X chromosome in a cell-type-specific fashion in *Drosophila*.	[85,86]

The best-studied candidate chromatin architectural lncRNA is XIST, which coats the entire inactive X-chromosome (Xi) in therian female mammals [59]. XIST exploits 3-D proximal contacts to spread throughout the entire chromosome and recruits machinery for catalyzing repressive chromatin modifications [60,61]. XIST acts both in transcription regulation as well as in the direct architectural stabilization of the native Xi structure [62–64]. Another well-studied X-chromosomal lncRNA, FIRRE, establishes contacts with several autosomes, suggesting that it might be involved in bridging these contact sites to bring them together [65]. HOTAIR is a lncRNA that binds to two distinct histone modifying complexes, PRC2 and LSD1/CoREST/REST complex at its 5′ domain 3′ ends, respectively and therefore in principle can bring H3K27 methylated and H3K4 unmethylated sites in close proximity [66]. A large number of euchromatic proteins also bind RNA that originates from loci in trans, suggesting a role for RNA in holding together two or more distant sites [67–70]. For example, NEAT1 and MATAL1 lncRNAs, which are components of paraspeckles and nuclear speckles, respectively, interact with transcripts from several euchromatic loci [69]. Similarly, enhancer RNAs are involved in chromatin interactions such as looping, although it remains unclear whether chromatin interactions are due to these RNAs themselves or are merely a consequence of enhancer activation [70–72]. Besides ncRNAs, nascent pre-mRNAs along with regulatory proteins may also play an active role in chromatin regulation and have been reviewed elsewhere [73].

Exact molecular mechanisms whereby lncRNAs might function as chromatin architectural elements remain speculative. Some lncRNAs such as HOTAIR that interact with two different chromatin modifying protein complexes, might act as bridges between two chromatin contact points [55]. RNA–RNA interactions are essential for maintaining ribonucleoprotein particles along with RNA–RBP interactions and can therefore play an important role in chromatin organization as well [74,75]. Such RNA–RNA interactions have been documented for XIST, which multimerizes via its A-repeat and circular RNAs that act as ‘sponges' for miRNAs [76–78].

## Role of RNA in CTCF-mediated chromatin organization

LncRNAs also impact the 3-D organization of mammalian genomes by facilitating the function of architectural proteins involved in chromatin looping. The multiple zinc-finger architectural protein CCCTC-binding factor (CTCF) acts as an insulator between facultative heterochromatin and euchromatin and organizes them into spatially disjoint domains [87]. CTCF also promotes interactions between distant genomic elements by mediating chromatin loop formation [24,88]. A small fraction of CTCF sites also occur at TAD boundaries, which prevent chromatin loops formed by loop extrusion factors (cohesin or condensin) from growing further [53,89,90]. CTCF contains an RNA-binding domain, exhibits high-affinity for specific RNAs (*K*_d_ < 1 nM) and is known to interact with thousands of transcripts (including XIST, TSIX, and XITE) [91,92]. CTCF RNA binding mutants show compromised self-association, binding to chromatin and chromatin loop formation, suggesting that CTCF-RNA interactions regulate chromatin looping [92–94].

## A possible role for architectural RNA in heterochromatin organization

Histone H3K9 methyl-marked constitutive heterochromatin is predominantly formed on repetitive DNA and is bound to the heterochromatin protein HP1 [95]. HP1 condenses *in vitro* reconstituted H3K9me-containing nucleosome arrays and bridges two H3K9me nucleosomes [96,97]. The ability to compact nucleosomal arrays of HP1 *in vitro* is only modest relative to the massive compaction at pericentric regions seen *in vivo*, suggesting that additional factors are required to condense H3K9me heterochromatin in the nucleus [96]. Interestingly, HP1 contains an RNA-binding domain and RNA binding is required for heterochromatin localization of mammalian HP1 to pericentric repetitive domains [98]. Drosophila HP1 is also known to interact with several RNAs originating mostly from repetitive regions [99]. In situ depletion of RNA leads to dispersion of H3K9me foci and compromises the ability of recombinant HP1 to bind to pericentric heterochromatin, raising the possibility that HP1 and RNA might act synergistically to compact chromatin [98]. Furthermore, specific depletion of pericentric major satellite (MajSat) transcripts with antisense oligonucleotides leads to a decrease in number of chromocenters (clusters of pericentric regions) per nucleus [100]. Chromocenters are also maintained by the RNA-binding SAF-B protein, which localizes to stress bodies and other locations as well [100]. These studies suggest that architectural RNAs together with RNA-binding proteins contribute to H3K9me heterochromatin compaction and possibly facilitate bridging between distant sites within megabase long heterochromatic domains. It is also possible that similar chromatin–RNA bridging interactions occur within domains of facultative heterochromatin compacted by the PRC1 complex, which is found to be associated with lncRNAs [101–103].

Components of membraneless RNA-filled nuclear bodies do not mix with the nucleoplasm because they undergo liquid-liquid phase separation (LLPS). Nuclear bodies, the best known examples of LLPS in the nucleus to date, phase-separate from the nucleoplasm due to multivalent weak interactions between RNA and RBPs [12,14,30,31,104,105]. The ability of RNA to form extensive secondary structures and establish multivalent interactions, and its net negative charge make RNA a potent modulator of LLPS. As a result, nuclear bodies such as the nucleolus and cytoplasmic P granules fulfil the most important criteria for classic LLPS behavior such as internal mixing, a critical concentration requirement, exchange of molecules with the nucleoplasm and ability to fuse with each other [29,105–108]. Other examples of RNA mediated LLPS are stress bodies that accumulate human pericentric repeat RNA (Sat III) [83,109]. RNA also buffers the phase separation of prion-like RBPs such that high RNA concentrations keep RBPs soluble and changes in RNA levels or RNA binding abilities of RBPs cause aberrant phase transitions [110].

Recent studies have attributed LLPS behavior to heterochromatin domains as well [111–114]. Components of both constitutive and facultative (HP1 and CBX2, respectively) form liquid droplets *in vitro* [111–115]. SAF-B also forms liquid droplets that are much more dynamic than those established by HP1 *in vitro*, and SAF-B knockdown results in a dispersed localization of pericentric heterochromatin suggesting that SAF-B-mediated phase separation may contribute to chromocenter condensates [100]. Unlike nuclear bodies, *in vivo* heterochromatin LLPS research is at an early stage and it remains to be determined how liquid droplets that form spontaneously from pure recombinant heterochromatin components *in vitro*, are regulated *in vivo* where one or more phase separating proteins (e.g. HP1 and SAF-B around pericentric regions) are present in a crowded environment of a given region. A recent investigation of biochemical and biophysical properties of *in vivo* HP1 foci showed a lack of LLPS behavior attributable to HP1 [116]. This suggests that the LLPS behavior of *in vivo* constitutive heterochromatin is much weaker when compared with nuclear bodies, and that the majority of the properties of heterochromatin can be explained by a classic condensed polymer model in which heterochromatin can be percolated with nucleoplasm.

**Figure 3.**
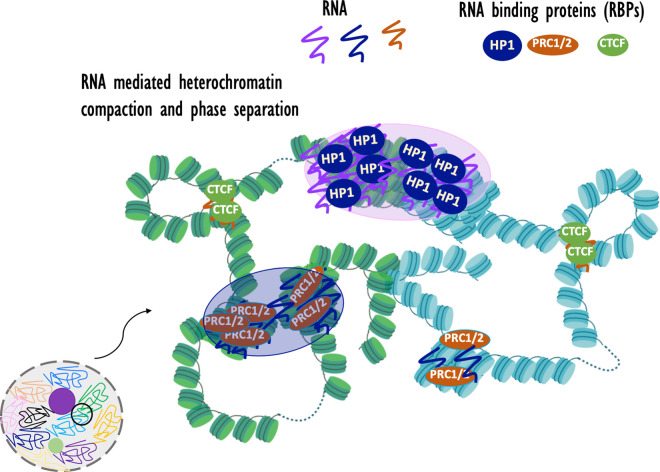
Architectural RNA and chromatin organization. Architectural RNAs may contribute to heterochromatin organization by facilitating compaction and possibly LLPS and to chromatin loop formation by tethering CTCF to distant loci. Nucleosomal arrays are shown from two different chromosomes that are marked in two different colors. Large transparent purple bubbles represent LLPS of compact heterochromatin.

To the extent that heterochromatin exhibits a degree of LLPS behavior, it is possible that architectural RNA is involved. HP1 and CBX2, respectively components of constitutive and facultative heterochromatin that compact nucleosome arrays, exhibit LLPS behavior *in vitro* and interact with several RNAs [96,111–113,117,118]. Recently, *in vitro* and *in vivo* LLPS behavior of HP1 and SAF-B was shown to be enhanced by pericentric MajSat RNA [100,119]. These initial reports hint at the possibility that heterochromatin LLPS involves multivalent weak RNA–RBP interactions (Figure 3).

## Perspectives

- The ability of RNA to participate in multivalent interactions via its sequence and secondary structures makes it an excellent architectural molecule, as is evident from its scaffolding role for nuclear bodies [31]. Architectural RNA may help to maintain heterochromatin compaction and centromeric chromatin structures. Architectural RNA–RBP interactions may also contribute to chromatin looping and intrachromosomal interactions in euchromatic regions.
- A next step in understanding the roles of architectural RNAs in maintaining chromatin structure is to apply genome-scale approaches that deplete specific RNAs from the intact nucleus *in situ* and that map RNA-chromatin interactions, such as GRID-seq, PIRCh-seq and CUT&RUN [120–122]. Characterization of specific chromatin architectural RNAs will set the stage for asking: 1) Which architectural RNA act in cis and which act in trans? 2) How exactly does a given architectural RNA make contact with the chromatin? Which one among RNA–RBP, RNA–RNA, or RNA–DNA (e.g. R-loops and G-quadruplexes) interactions are used by a given architectural lncRNA?
- Understanding the role of RNA mediated compaction in the formation of heterochromatic foci such as chromocenters in the mouse nucleus should shed light on the possible role of LLPS in heterochromatin condensation.
- Finally, RNA components of heterochromatin assemblies might provide sequence specificity to RBPs in heterochromatin, including CENPs, HP1, SAF-B and PRC1, and understanding the mechanistic basis for these interactions is an important goal of chromatin research.

## Abbreviations

CTCF: CCCTC-binding factor
HP1: heterochromatin protein 1
LLPS: liquid-liquid phase separation
PRC1: Polycomb Repressive Complex 1
RBP: RNA binding protein
TAD: topologically associated domain
